# Potential and Safety Assessment of a Hydrogel as a Vitreous Substitute Following Vitrectomy in an Ex Vivo Porcine Model

**DOI:** 10.3390/gels12100900

**Published:** 2026-10-07

**Authors:** Soheil Sojdeh, Zahra Bibak Bejandi, Miranda Castillo, Gregory Tsougranis, Anwar N. Khandaker, R. V. Paul Chan, Zohreh Arabpour, Ali R. Djalilian

**Affiliations:** Department of Ophthalmology and Visual Science, University of Illinois, Chicago, IL 60612, USA; sojdesoheil@gmail.com (S.S.); gtsoug2@uic.edu (G.T.); kanwar@uic.edu (A.N.K.); rvpchan@uic.edu (R.V.P.C.)

**Keywords:** hyaluronic acid, PEG-Tyramine, vitreous substitute, hydrogel, vitrectomy, rheology, optical transparency, cytocompatibility, ocular biomaterials, ex vivo porcine model

## Abstract

Purpose: To develop and preliminarily evaluate a hyaluronic acid–1,4-butanediol diglycidyl ether (HA-BDDE)/polyethylene glycol–tyramine (PEG-Tyramine) dual-hydrogel designed as a transparent and injectable vitreous substitute with suitable optical, rheological, and cytocompatibility characteristics for potential application following vitrectomy. Methods: HA-BDDE and PEG-Tyramine were prepared separately and combined to form a dual-hydrogel formulation. The resulting hydrogel was characterized using attenuated total reflectance Fourier-transform infrared (ATR-FTIR) spectroscopy, proton nuclear magnetic resonance (^1^H NMR) spectroscopy, oscillatory rheometry, and spectrophotometry to evaluate its chemical, rheological, and optical properties. Cytocompatibility was assessed using retinal pigment epithelial (RPE) cells through the methyl thiazolyl tetrazolium (MTT) assay and Live/Dead fluorescence staining. Ex vivo performance was evaluated following hydrogel injection into porcine eyes after vitrectomy, with assessment of macroscopic transparency, structural integrity, and retention within the vitreous cavity. Results: The HA-BDDE/PEG-Tyramine formulation formed a transparent and macroscopically homogeneous hydrogel phase. Spectroscopic analyses demonstrated the characteristic chemical functionalities and molecular environments associated with the HA- and PEG-containing components. Rheological analysis showed predominantly elastic, gel-like behavior, with the storage modules exceeding the loss modulus across the investigated frequency range. The hydrogel exhibited measurable and relatively stable visible-light transmission across the evaluated wavelength range. RPE cells maintained high metabolic activity during the culture period, while Live/Dead imaging demonstrated predominantly viable cells with limited dead-cell staining. In the ex vivo porcine model, the hydrogel remained within the vitreous cavity and maintained its macroscopic transparency and structural integrity during the observation period. Conclusions: The HA-BDDE/PEG-Tyramine dual-hydrogel exhibited favorable optical, rheological, cytocompatibility, and short-term ex vivo retention characteristics. These findings support further investigation of this dual-hydrogel system as a potential injectable vitreous substitute following vitrectomy.

## 1. Introduction

The vitreous body is a transparent, viscoelastic extracellular matrix that occupies the posterior segment of the eye and contributes to ocular structure, optical clarity, and the biomechanical environment of the retina TT Kleinberg et al. [1]. It consists primarily of water, with hyaluronic acid (HA), collagen, proteoglycans, and other extracellular matrix components contributing to its characteristic viscoelastic properties [2,3]. Maintaining an appropriate vitreous environment is therefore important for preserving the structural and optical functions of the posterior segment. Pars plana vitrectomy is widely used for the treatment of various vitreoretinal disorders, including retinal detachment, vitreous hemorrhage, epiretinal membrane, and macular hole. Following removal of the native vitreous, the vitreous cavity is commonly filled with balanced salt solution, gas, or silicone oil depending on the surgical indication [4]. Although these materials are clinically useful and remain essential in vitreoretinal surgery, they do not fully reproduce the biochemical, optical, and viscoelastic characteristics of the native vitreous [1,5,6]. The development of an ideal vitreous substitute has therefore remained an active area of research for several decades, encompassing conventional tamponade agents as well as natural, synthetic, and hybrid biomaterials. Comprehensive reviews have highlighted the major requirements for vitreous replacement, including optical transparency, appropriate viscoelasticity, biocompatibility, injectability, structural stability, and long-term intraocular performance. More recent reviews have particularly emphasized hydrogel-based systems as promising candidates because their water-rich polymer networks can be engineered to provide tunable mechanical, optical, and biological properties while allowing injectable or in situ-forming strategies [4,7].

Hydrogels are attractive candidates for vitreous replacement because their highly hydrated three-dimensional polymer networks can be engineered to provide tunable mechanical and viscoelastic properties while maintaining optical transparency [8,9]. An ideal vitreous substitute should exhibit adequate transparency, appropriate viscoelasticity, structural stability, injectability, and biocompatibility while remaining sufficiently stable within the vitreous cavity [8]. For a vitreous substitute, these properties must be appropriately balanced to provide sufficient structural organization and viscoelasticity while maintaining optical transparency and allowing controlled administration into the vitreous cavity [10].

Hyaluronic acid is particularly relevant to vitreous substitute development because it is a major glycosaminoglycan present in the native vitreous and contributes to its hydration and viscoelastic behavior [6,7]. However, native HA can undergo relatively rapid degradation and may not provide sufficient structural stability when used alone. Chemical crosslinking can improve the stability and mechanical properties of HA-based hydrogels while preserving their highly hydrated nature [4,11]. 1,4-Butanediol diglycidyl ether (BDDE) is an epoxide-based crosslinking agent that has been used to generate chemically crosslinked HA networks with improved structural stability [12,13].

Poly(ethylene glycol) (PEG) is another widely investigated biomaterial because of its hydrophilicity, tunable physicochemical properties, and favorable biocompatibility [10]. Functionalization of PEG with tyramine introduces tyramine-derived aromatic and phenolic functionalities into the polymer structure, which may contribute to the physicochemical characteristics and intermolecular interactions of the resulting hydrogel system [13]. Thus, HA- and PEG-based materials offer complementary characteristics that may be relevant to vitreous substitution. HA provides a highly hydrated matrix with biological relevance to the native vitreous, whereas PEG-based systems provide a hydrophilic and tunable polymer platform that can contribute to the physicochemical and structural properties of the hydrogel. Combining these two polymer components may therefore provide a means of balancing optical transparency, hydration, viscoelastic behavior, structural stability, and biocompatibility rather than relying on a single polymer system to provide all of these characteristics [14,15].

In this study, we developed a dual-hydrogel composed of HA-BDDE and PEG-Tyramine. The resulting formulation was characterized using spectroscopic, rheological, and optical analyses to evaluate its chemical composition, viscoelastic properties, and transparency. Cytocompatibility was assessed using retinal pigment epithelial cells, while ex vivo evaluation in a porcine vitrectomy model was used to assess hydrogel retention, transparency, and structural integrity within the vitreous cavity. The formulation was designed based on the complementary properties of the two polymer components, with the aim of developing a hydrated and optically transparent material with suitable viscoelastic and structural characteristics for vitreous replacement. Collectively, these evaluations were designed to provide an initial preclinical assessment of the potential of the HA-BDDE/PEG-Tyramine dual-hydrogel as an injectable vitreous substitute.

The rationale for combining HA-BDDE and PEG-Tyramine was to exploit the complementary physicochemical characteristics of two hydrophilic polymeric components rather than to simply generate a second hydrogel from two pre-existing hydrogels. HA-BDDE provides a highly hydrated, HA-based network with structural stabilization through BDDE crosslinking, while PEG-Tyramine provides an additional hydrophilic polymeric component with chemically introduced tyramine functionalities. Their combination was therefore designed as a formulation strategy to modulate the overall hydration, viscoelastic response, and physicochemical characteristics of the resulting material. Importantly, the present study does not assume that the two preformed networks undergo complete covalent inter-network crosslinking; rather, the final formulation is considered a physically combined dual-hydrogel system whose properties arise from the contributions and interactions of both components.

## 2. Results and Discussion

### 2.1. Physicochemical and Structural Characterization of the HA-BDDE/PEG-Tyramine Dual-Hydrogel

**Figure 1:**
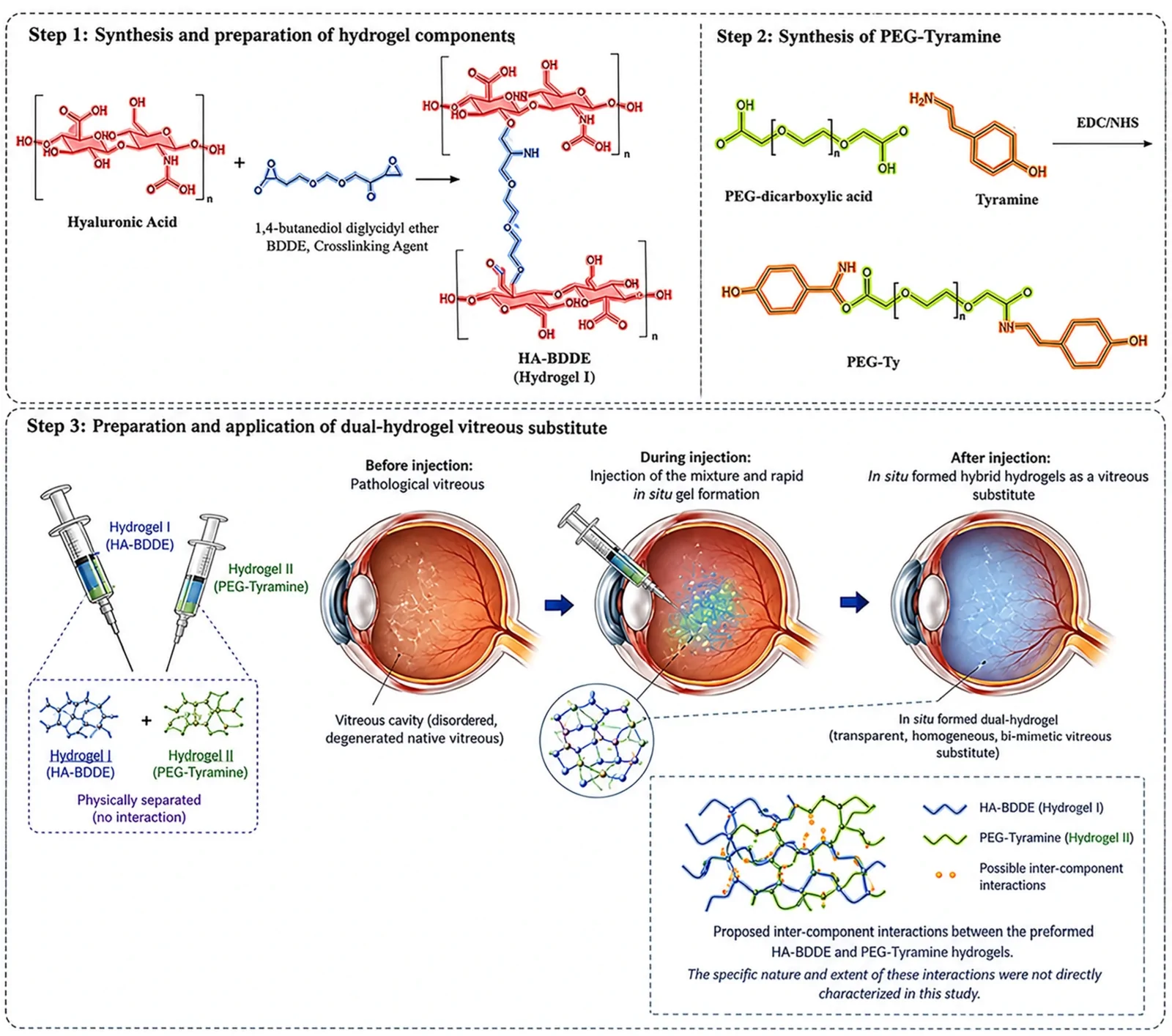
Schematic illustration of the preparation and formation of the HA-BDDE/PEG-Tyramine dual-hydrogel vitreous substitute. HA-BDDE (Hydrogel I) and PEG-Tyramine (Hydrogel II) were synthesized, purified, and prepared as separate hydrogel components prior to use. The preformed components were stored separately and combined at a predetermined 4:1 (*w*/*w*) ratio immediately before administration. The lower schematic illustrates possible inter-component interactions between the two preformed hydrogel components. The specific nature and extent of these interactions were not directly characterized in the present study.

The HA-BDDE/PEG-Tyramine dual-hydrogel was prepared using a two-component formulation comprising HA-BDDE (Hydrogel I) and PEG-Tyramine (Hydrogel II) at a 4:1 (*w*/*w*) ratio. As illustrated in Figure 1, HA was crosslinked with 1,4-butanediol diglycidyl ether (BDDE) to form the HA-BDDE network, while PEG-dicarboxylic acid was conjugated with tyramine using EDC/NHS chemistry to obtain the PEG-Tyramine component. The two hydrogel components were maintained as physically separated phases prior to administration and subsequently combined during application, resulting in a visually homogeneous hydrogel phase.

The design of the present vitreous substitute was based on the combination of two independently prepared hydrogel components, HA-BDDE and PEG-Tyramine. This dual-component strategy was designed to combine the highly hydrated and biologically relevant characteristics of HA with the hydrophilic and flexible nature of PEG while introducing complementary chemical functionalities through BDDE and tyramine. HA is particularly relevant to vitreous substitution because it is a major component of the native vitreous and contributes to its hydrated and viscoelastic character. PEG-based materials, in contrast, provide a synthetic platform with tunable physicochemical properties and have been investigated in hydrogel systems intended for vitreous replacement [16]. The combination of these two polymeric components therefore provides a rational materials-design approach for integrating complementary characteristics within a single vitreous substitute [14]. Rather than relying on a single precursor formulation, the two components in the present system were prepared separately and maintained as physically distinct phases before administration. This configuration provides a practical approach for handling and administration while allowing the two components to interact after being brought into contact. From a practical standpoint, this two-component configuration does not require synthesis, purification, or crosslinking immediately before surgery, as both hydrogel components can be prepared and processed in advance. The final preparation step is limited to combining the preformed components at the predetermined ratio immediately before administration.

The molecular basis for the interaction between the preformed HA-BDDE and PEG-Tyramine hydrogel components requires careful consideration. BDDE is a bifunctional epoxide crosslinker that reacts with nucleophilic groups in HA during formation of the HA-BDDE network [8,9]. Epoxide–amine reactions are well established in organic and polymer chemistry and generally proceed through nucleophilic ring opening of the epoxide, producing a β-amino alcohol functionality [17,18]. Primary amines can undergo successive reactions with epoxide groups, while hydroxyl groups generated during epoxide ring opening may further influence the reaction through hydrogen bonding and autocatalytic effects [18,19].

However, the application of this classical epoxide–amine mechanism to the present HA-BDDE/PEG-Tyramine system should be considered with caution. In the present synthesis, tyramine was conjugated to carboxyl-terminated PEG through EDC/NHS-mediated amide coupling. Therefore, the amine functionality of tyramine is expected to be consumed during amide-bond formation, and the present study did not directly quantify residual free amine groups or residual epoxide groups after purification. Accordingly, a direct covalent epoxide–amine coupling between the two preformed hydrogel networks cannot be established from the current experimental data.

A possible contribution from residual reactive functionalities cannot nevertheless be completely excluded. In particular, residual epoxide groups within the HA-BDDE network, if present, could potentially interact with nucleophilic or hydroxyl-containing functionalities associated with the PEG-Tyramine component under appropriate conditions. Phenolic hydroxyl–epoxide ring-opening reactions have been reported in epoxy/phenolic systems, although such reactions depend strongly on the reaction conditions and can be facilitated by appropriate catalysts [20].

Therefore, the visually homogeneous appearance observed after combining the two preformed components should not be interpreted as direct evidence of covalent inter-network crosslinking. The observed integration may arise from a combination of physical interpenetration, hydrogen bonding, chain entanglement, and, potentially, limited chemical interactions involving residual reactive groups. The relative contribution of these mechanisms remains unresolved in the present study. Direct quantification of residual epoxide and free amine/phenolic functionalities, together with complementary spectroscopic or spectrometric analysis before and after mixing, would be required to establish whether new covalent inter-network bonds are formed.

The present dual-hydrogel strategy differs from previously reported PEG-based in situ-forming vitreous substitutes. Wang et al. developed a PEG-based zwitterionic hydrogel (PEGBAA) designed to undergo rapid in situ gelation and provide antifouling properties, with subsequent evaluation in a rabbit model for long-term vitreous replacement [10]. In contrast, the present study focuses on a preformed dual-hydrogel system comprising HA-BDDE and PEG-Tyramine, with the two components prepared separately and combined at a predetermined ratio. The primary objective of the present work was to establish the physicochemical characteristics, optical behavior, RPE cytocompatibility, and short-term ex vivo retention of this formulation rather than to reproduce the in situ-forming and long-term antifouling strategy reported previously.

The use of two preformed hydrogel components was intended to provide complementary contributions to the final formulation. HA-BDDE was selected as the primary hydrated network because HA is an important component of the native vitreous extracellular matrix and can provide a highly hydrated environment, while BDDE crosslinking provides structural stabilization. PEG-Tyramine was incorporated as a second hydrophilic polymeric component to provide additional control over the physicochemical characteristics of the formulation. Thus, the purpose of combining the two components was not to imply that either component was incapable of forming a hydrogel independently, but rather to formulate a material in which the contributions of both components could be combined in a single injectable vitreous-substitute formulation.

### 2.2. FTIR and NMR Characterization

**Figure 2:**
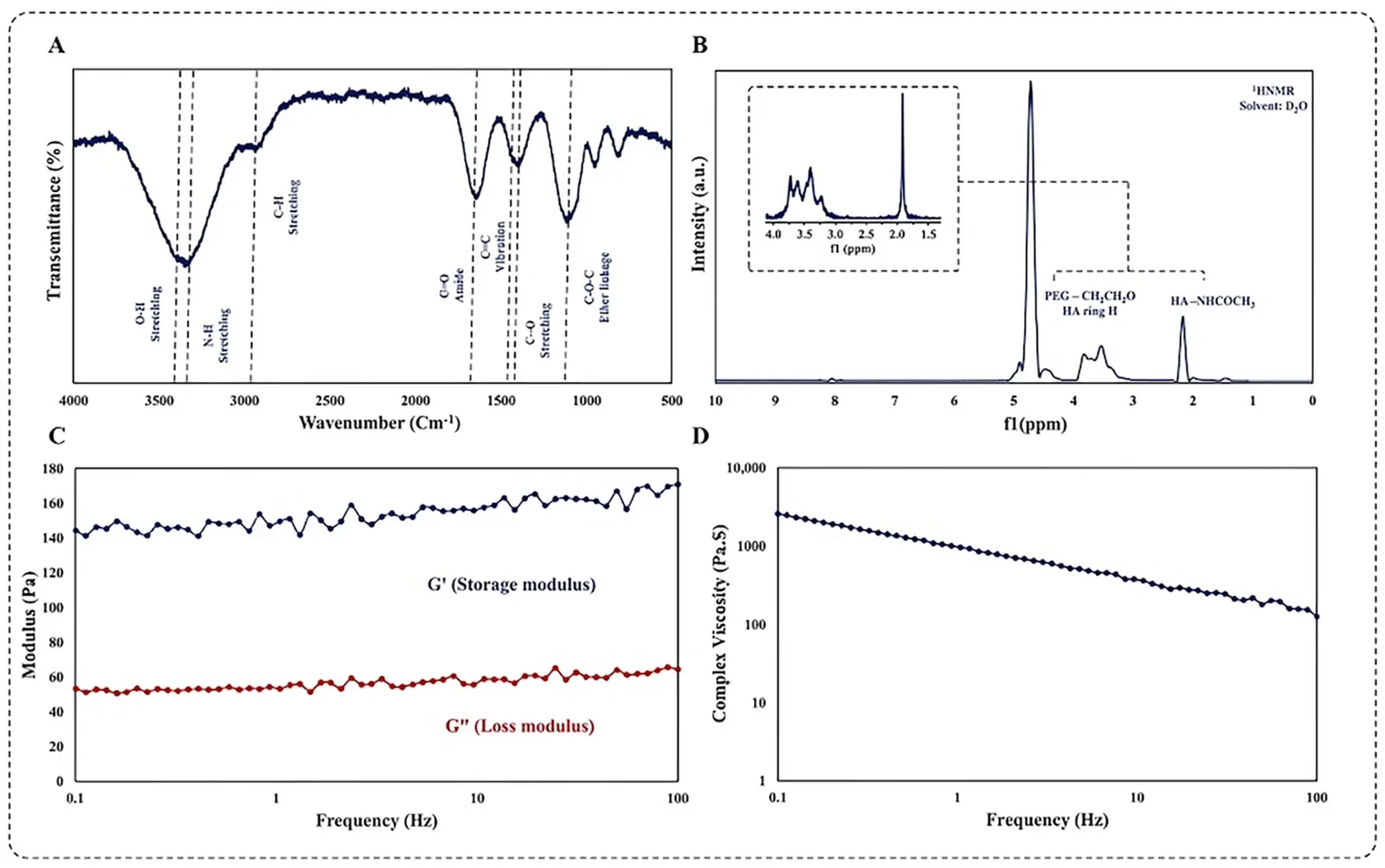
Physicochemical and rheological characterization of the HA-BDDE/PEG-Tyramine dual-hydrogel. (**A**) ATR-FTIR spectrum shows the characteristic functional groups of the hydrogel. (**B**) 1H NMR spectrum recorded in D_2_O, showing characteristic proton environments associated with the PEG- and HA-containing components and detectable aromatic signals attributable to the tyramine-derived moiety. (**C**) Frequency-dependent storage modulus (G′) and loss modulus (G″) over 0.1–100 Hz, demonstrating the predominantly elastic behavior of the hydrogel (G′ > G″). (**D**) Complex viscosity as a function of frequency, showing a pronounced frequency-dependent decrease in viscosity.

The chemical characteristics of the final HA-BDDE/PEG-Tyramine dual-hydrogel were investigated using ATR-FTIR and 1H NMR spectroscopy. As shown in Figure 2A, the ATR-FTIR spectrum exhibited a broad absorption band centered at approximately 3400 cm^−1^, attributable to overlapping O-H and N-H stretching vibrations. The absorption features in the ~2900–3000 cm^−1^ region were assigned to C-H stretching vibrations. The band near ~1650 cm^−1^ was associated with amide C=O stretching, while the feature around ~1550 cm^−1^ was consistent with amide-related vibrations. The absorption in the ~1400 cm^−1^ region was attributed to COO^−^ stretching, while the bands in the ~1000–1150 cm^−1^ region were associated with C-O-C stretching vibrations. Overall, the observed FTIR features were consistent with the characteristic functional groups expected for the HA- and PEG-containing hydrogel system.

The molecular characteristics of the final hydrogel were further examined by 1H NMR spectroscopy in D_2_O (Figure 2B). The strong resonance at approximately 4.7 ppm was attributed predominantly to residual H_2_O/HOD in the deuterated aqueous solvent. The resonance envelope in the ~3.4–4.0 ppm region was associated with overlapping proton environments from the PEG -CH_2_CH_2_-O- backbone and HA repeating units, whereas the signal near ~2.0 ppm was attributed to methyl protons of the N-acetyl groups of HA. Collectively, the FTIR and 1H NMR profiles were consistent with the expected chemical functionalities and molecular environments of the final HA-BDDE/PEG-Tyramine dual-hydrogel.

The spectroscopic findings provide complementary evidence for the chemical composition of the final material. The FTIR profile displayed the characteristic hydroxyl-, amide-, carboxylate-, and ether-containing functionalities expected from the HA- and PEG-containing formulation. Similarly, the 1H NMR spectrum demonstrated proton environments associated with the HA- and PEG-containing components, together with detectable aromatic environments attributable to the tyramine-derived component. The simultaneous presence of these characteristic chemical environments indicates that the principal molecular features of the constituent materials were retained following formation of the final dual-hydrogel. Importantly, these spectroscopic observations primarily support the expected chemical composition of the formulation and do not independently establish the extent of inter-network covalent coupling. Thus, the FTIR and NMR findings are best interpreted as evidence consistent with successful formation of the intended HA-BDDE/PEG-Tyramine material rather than direct proof of the proposed epoxide-amine reaction [21].

### 2.3. Rheological Properties

The viscoelastic behavior of the HA-BDDE/PEG-Tyramine hydrogel was evaluated by frequency sweep analysis over 0.1–100 Hz (Figure 2C). Throughout the investigated frequency range, the storage modulus (G′) remained higher than the loss modulus (G″). G′ increased from approximately 140–150 Pa at low frequencies to approximately 165–170 Pa at the highest frequency, whereas G″ remained lower and increased from approximately 50–55 Pa to approximately 60–65 Pa. The persistent predominance of G′ over G″ indicates a predominantly elastic, gel-like response throughout the investigated frequency range. No crossover between G′ and G″ was observed. The complex viscosity (η*) exhibited a pronounced frequency-dependent decrease (Figure 2D), declining from approximately 2.5 × 10^3^ Pa·s at 0.1 Hz to approximately 1.3 × 10^2^ Pa·s at 100 Hz. This decrease indicates progressively lower resistance to dynamic deformation with increasing frequency. Overall, the hydrogel maintained a predominantly elastic character while exhibiting a pronounced frequency dependence of complex viscosity across the investigated frequency range.

The rheological behavior provides an important indication of the structural character of the resulting hydrogel. However, because rheological measurements of the individual HA-BDDE and PEG-Tyramine components and appropriate formulation controls were not included in the present study, the observed viscoelastic response cannot be used to distinguish covalent inter-component crosslinking from the combined contributions of the individual hydrogel components and their physical interactions. The persistent predominance of the storage modulus over the loss modulus across the investigated frequency range indicates that the material exhibits an elastic-dominant, gel-like response rather than behaving predominantly as a viscous liquid. This behavior is relevant to vitreous substitution because the material must maintain a continuous three-dimensional structure after placement within the vitreous cavity rather than behaving as a freely flowing liquid. The absence of a crossover between the storage and loss moduli further indicates that the elastic contribution remained dominant over the dynamic timescales investigated [22]. Such behavior is consistent with the formation of a mechanically coherent hydrated polymer network and supports the potential of the formulation to maintain its structural organization after administration.

The frequency dependence of complex viscosity provides complementary information regarding the dynamic response of the hydrogel. The decrease in complex viscosity with increasing frequency indicates that the resistance of the material to oscillatory deformation is dependent on the timescale of applied deformation. From a materials-design perspective, such frequency dependence may be advantageous for an injectable vitreous substitute because the material should be capable of undergoing deformation during handling and administration while retaining sufficient structural character under less rapid deformation after placement [8]. Nevertheless, these measurements were obtained using oscillatory frequency-sweep conditions and therefore characterize the frequency-dependent viscoelastic response of the material rather than its steady-shear flow behavior. Accordingly, the present rheological data should not be interpreted as direct evidence of shear-thinning. Dedicated steady-shear measurements would be required to establish the flow behavior of the hydrogel under controlled shear conditions.

### 2.4. Optical Transparency

**Figure 3:**
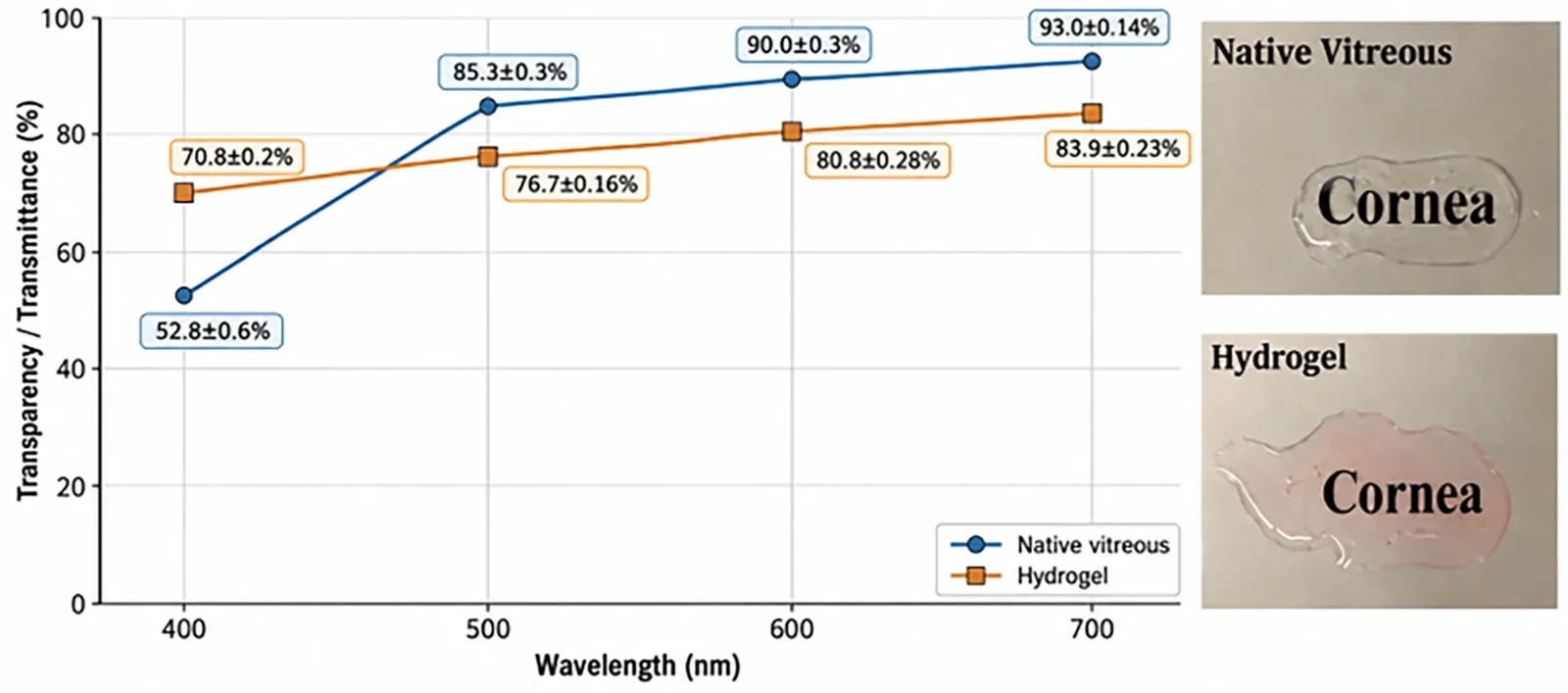
Optical transmittance spectra of native porcine vitreous and the hydrogel substitute across the visible-light wavelength range (400–700 nm).

To evaluate the optical properties of the hydrogel-based vitreous substitute, its optical transmittance was determined by spectrophotometric analysis at selected wavelengths within the visible-light range of 400–700 nm and compared with that of native porcine vitreous (Figure 3). Measurements were performed using a Molecular Devices SpectraMax iD microplate reader (Thermo Fisher Scientific, Waltham, MA , USA) at 400, 500, 600, and 700 nm under identical experimental conditions. Five independent samples of each material were analyzed, with 100 µL of sample loaded into individual wells of a 96-well plate. A blank was used as the 100% transmittance reference.

The native vitreous exhibited mean transmittance values of 52.8 ± 0.6%, 85.38 ± 0.30%, 90.00 ± 0.33%, and 93.07 ± 0.14% at 400, 500, 600, and 700 nm, respectively. The hydrogel exhibited mean transmittance values of 70.8 ± 0.2%, 76.78 ± 0.16%, 80.81 ± 0.28%, and 83.90 ± 0.23% at the corresponding wavelengths. Data are presented as mean ± standard deviation (SD), and error bars representing the SD are shown in Figure 3.

Both native vitreous and the hydrogel exhibited increasing transmittance with increasing wavelength. At 400 nm, the hydrogel exhibited higher transmittance than native vitreous (70.8 ± 0.2% vs. 52.8 ± 0.6%). In contrast, at 500, 600, and 700 nm, native vitreous exhibited higher transmittance than the hydrogel, with values of 85.38 ± 0.30%, 90.00 ± 0.33%, and 93.07 ± 0.14%, respectively, compared with 76.78 ± 0.16%, 80.81 ± 0.28%, and 83.90 ± 0.23% for the hydrogel. The hydrogel therefore demonstrated substantial and progressively increasing visible-light transmission across the investigated wavelength range. Although the hydrogel exhibited lower transmittance than native vitreous at wavelengths of 500–700 nm, qualitative examination showed that the hydrogel was macroscopically transparent and allowed visualization through the material. The observed transmission profile indicates that the hydrogel retained measurable optical transmission throughout the visible-light range, supporting its potential suitability for applications requiring light transmission within the vitreous cavity. Optical performance is another critical consideration for a material intended to replace the vitreous body. Because the native vitreous provides an optically clear pathway between the lens and retina, a candidate substitute must retain sufficient visible-light transmission following hydrogel formation [23].

In the present study, the hydrogel remained optically transparent at the macroscopic level and exhibited measurable transmission throughout the 400–700 nm wavelength range. The measurements obtained using the calibrated Molecular Devices SpectraMax iD reader showed that hydrogel transmittance increased from 70.8 ± 0.2% at 400 nm to 83.90 ± 0.23% at 700 nm. At wavelengths of 500–700 nm, the hydrogel exhibited lower transmittance than native porcine vitreous, although the difference remained relatively consistent across this range. This wavelength-dependent behavior is relevant to the optical assessment of the material because the visible-light transmission of the hydrogel was not uniformly equivalent to that of native vitreous. Nevertheless, the hydrogel maintained substantial measurable transmission across the investigated visible-light range and did not exhibit an abrupt loss of transmission between the measured wavelengths. This observation is important because excessive turbidity, phase separation, or opacity could compromise the optical function expected from a vitreous substitute. The present results therefore demonstrate that the formulation possesses measurable visible-light transmission, while the difference from native vitreous indicates that further optical optimization may be warranted [22,23].

The combined rheological and optical findings highlight an important aspect of vitreous-substitute design: the material must simultaneously maintain structural organization and permit optical transmission. These requirements can be difficult to achieve simultaneously because increasing polymer concentration or crosslinking density may improve mechanical stability while potentially affecting optical clarity and handling characteristics [22]. The present dual-hydrogel exhibited an elastic-dominant response together with measurable visible-light transmission, indicating that the selected formulation provides several fundamental physicochemical characteristics relevant to vitreous replacement. However, these results should not be interpreted as demonstrating complete physicochemical equivalence to native vitreous. Native vitreous possesses a highly complex extracellular matrix architecture, and properties such as long-term mechanical behavior, degradation, hydration, refractive characteristics, and response to intraocular physiological conditions cannot be established from the present measurements alone [24]. Rather, the current findings provide a material-property basis for further evaluation of the hydrogel as a potential vitreous substitute.

Overall, the optical measurements demonstrate that the HA-BDDE/PEG-Tyramine hydrogel possesses substantial visible-light transmittance across the 400–700 nm range. While its transmittance was lower than that of native porcine vitreous at 500–700 nm, the hydrogel remained macroscopically transparent and exhibited a progressive increase in light transmission with increasing wavelength. These findings support the optical potential of the hydrogel as a vitreous substitute while indicating that further optimization of formulation composition, crosslinking, and network homogeneity may be beneficial for improving its optical properties and more closely approximating those of native vitreous.

### 2.5. Cytocompatibility by Live/Dead and MTT Assays

**Figure 4:**
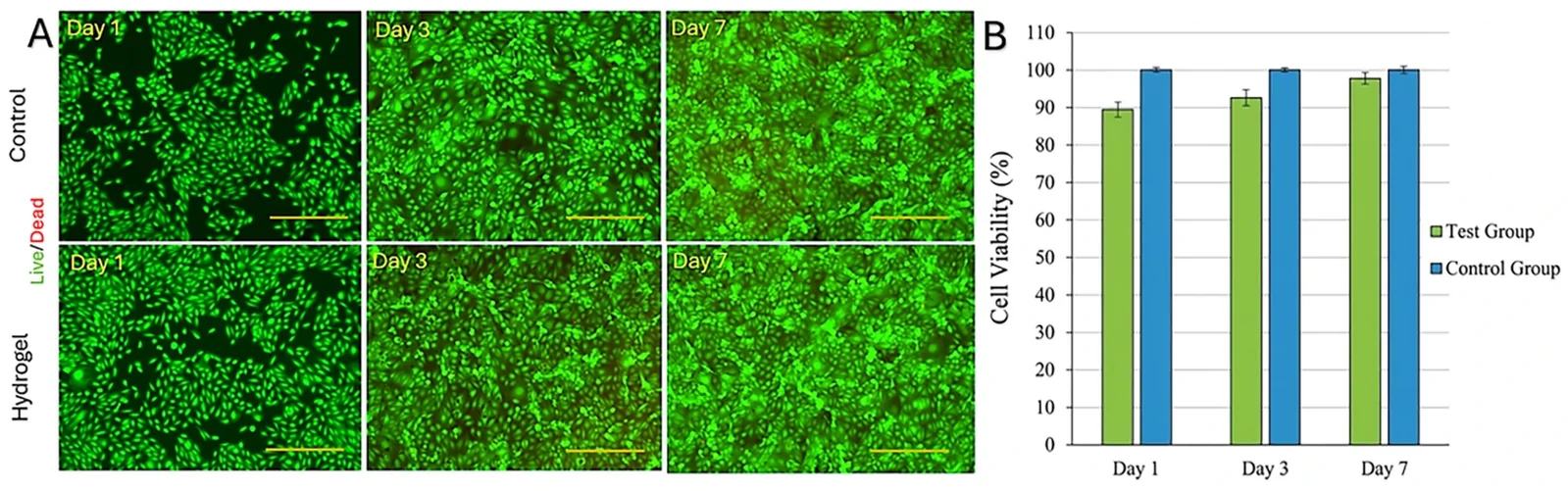
Cytocompatibility assessment of the hydrogel using RPE cells. (**A**) Representative Live/Dead fluorescence images at Days 1, 3, and 7; viable cells emit green fluorescence and dead cells emit red fluorescence (scale bar: 250 µm). (**B**) Relative cell viability measured by the MTT assay at Days 1, 3, and 7, expressed as a percentage of the tissue-culture-plate control.

Live/Dead fluorescence imaging provided additional qualitative evidence of cellular compatibility. At Days 1, 3, and 7, RPE cells cultured on the hydrogel demonstrated predominantly green fluorescence, indicative of viable cells, with limited red PI-positive cells. The overall distribution and morphology of viable cells were comparable to those observed in the hydrogel-free control group. No substantial increase in dead-cell staining was observed over the 7-day observation period, further supporting cytocompatibility of the hydrogel (Figure 4A).

The cytocompatibility of the hydrogel was further evaluated using an MTT assay after 1, 3, and 7 days of culture with RPE cells. Cell viability was expressed relative to the tissue-culture-plate control, which was defined as 100%. The hydrogel exhibited relative cell viabilities of 90.43%, 92.16%, and 97.21% at Days 1, 3, and 7, respectively. Cell viability increased progressively over the 7-day culture period, reaching approximately 97% of the control level by Day 7. Overall, the MTT results demonstrated that exposure to the hydrogel maintained high cell viability throughout the 7-day culture period, with no apparent progressive cytotoxic effect (Figure 4B).

The cytocompatibility findings provide additional support for the biological feasibility of the hydrogel under the tested in vitro conditions. The sustained metabolic activity observed in the MTT assay, together with the predominantly viable-cell pattern observed by Live/Dead fluorescence imaging, indicates that exposure to the hydrogel did not produce an obvious progressive cytotoxic response during the culture period. The agreement between the two complementary assays is particularly relevant because MTT provides an indirect measure of cellular metabolic activity, whereas Live/Dead staining provides qualitative information regarding cellular viability. Together, these findings support the compatibility of the hydrogel with RPE cells under the experimental conditions investigated. Nevertheless, in vitro cytocompatibility with RPE cells cannot be considered equivalent to complete intravitreal biocompatibility. The intraocular environment involves multiple retinal and ocular cell populations as well as inflammatory, enzymatic, and mechanical factors that are not reproduced in the present cell-culture model. Further evaluation involving retinal histology, inflammatory markers, and retinal functional assessments will therefore be necessary.

### 2.6. Ex Vivo Evaluation of Hydrogel Vitreous Substitute in a Porcine Vitrectomy Model

**Figure 5:**
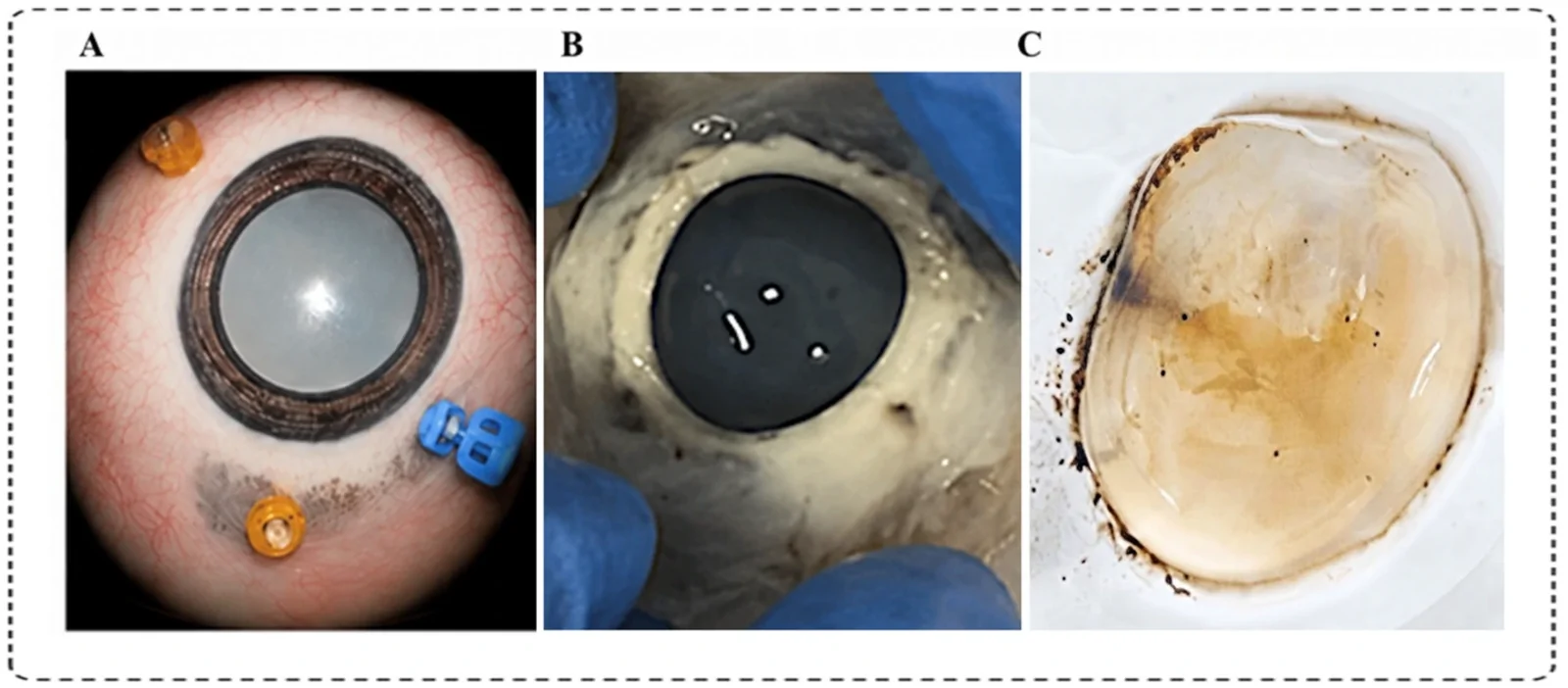
Ex vivo evaluation of the injectable HA-BDDE/PEG-Tyramine dual-hydrogel as a vitreous substitute in a porcine eye model. (**A**) Porcine eye following pars plana vitrectomy. (**B**) Gross appearance of the eye one week after hydrogel administration. (**C**) Retrieved vitreous substitute after one week, demonstrating retention and maintenance of its macroscopic integrity and transparency within the vitreous cavity.

The hydrogel demonstrated good injectability and ease of handling during intraocular administration through a 23-gauge needle following ex vivo vitrectomy. After hydrogel injection and sealing of the sclerotomy sites, the porcine globes were maintained in DMEM at room temperature for 1 week. Throughout the observation period, the globes retained their overall structural integrity. At the end of the 1-week observation period, the external morphology and overall shape of the porcine globes appeared normal, with no apparent gross deformation, collapse, or other visible structural abnormalities (Figure 5B). Upon removal from the vitreous cavity, the hydrogel maintained its overall appearance and cohesive structure, with no obvious gross changes in morphology (Figure 5C). These findings demonstrate that the hydrogel remained grossly stable and retained its structural integrity within the vitreous cavity throughout the 1-week ex vivo observation period.

The ex vivo porcine eye experiments further support the practical feasibility of the formulation following vitrectomy. Following administration through a small-gauge cannula, the hydrogel remained within the vitreous cavity during the observation period and retained a macroscopically transparent and viscous appearance. The absence of obvious gross fragmentation or loss of structural integrity suggests that the material can maintain a continuous phase after placement within the surgically altered vitreous cavity. This observation is particularly relevant to the intended application because a vitreous substitute must not only be injectable but also remain sufficiently organized within the cavity after administration. The ex vivo findings therefore provide an important bridge between characterization of the hydrogel as a material and its practical behavior within a surgically manipulated ocular environment [4].

The retention observed in the ex vivo model should, however, be interpreted as evidence of short-term physical feasibility rather than long-term intraocular stability. An isolated porcine globe cannot reproduce the fluid turnover, intraocular pressure, enzymatic activity, inflammatory responses, cellular interactions, and other physiological processes present in a living eye. In addition, the limited observation period does not establish long-term persistence, degradation, volume stability, or maintenance of optical properties. Consequently, the ex vivo findings demonstrate that the hydrogel can remain macroscopically associated with the vitreous cavity over the investigated period, but they cannot establish its long-term behavior under physiological intraocular conditions. These questions will require appropriately designed in vivo intravitreal studies.

Several limitations should be considered when interpreting the present findings. First, the ex vivo porcine model provides a useful platform for assessing administration, retention, transparency, and gross structural behavior but does not reproduce the complete biological environment of a living eye. In particular, the present study did not include in vivo intravitreal administration or long-term ocular evaluation; therefore, intraocular retention, degradation, inflammatory responses, retinal safety, and functional effects under physiological conditions remain to be established.

Second, the present rheological characterization was based on oscillatory frequency-sweep measurements; therefore, steady-shear flow behavior and shear-thinning were not directly established. In addition, the rheological measurements do not fully capture the mechanical behavior of the hydrogel under the dynamic and prolonged loading conditions encountered within the vitreous cavity. Third, although the homogeneous appearance of the mixed hydrogel is consistent with physical integration of the two components, the specific molecular basis of their inter-component interactions was not directly quantified. Residual epoxide and free amine/phenolic functionalities, as well as newly formed inter-component covalent bonds, were not directly measured. Therefore, the current data do not establish covalent inter-network coupling, and the observed integration may involve physical interpenetration, hydrogen bonding, chain entanglement, and/or limited chemical interactions. Direct chemical and physicochemical analyses, including residual functional-group quantification, swelling ratio, gel fraction, and comparative formulation controls, will be required to further elucidate the network structure. Future studies incorporating in vivo intravitreal evaluation, extended degradation and stability studies, broader ocular biocompatibility assessments, and clinically relevant injection and mechanical testing will be important to determine the long-term performance and translational potential of the HA-BDDE/PEG-Tyramine dual-hydrogel.

## 3. Conclusions

The present study provides a preliminary physicochemical, biological, and ex vivo evaluation of an HA-BDDE/PEG-Tyramine dual-hydrogel developed as an injectable vitreous substitute. The material exhibited the expected chemical characteristics, predominantly elastic viscoelastic behavior, measurable visible-light transmission, favorable short-term compatibility with RPE cells, and retention with preserved macroscopic integrity within an ex vivo porcine vitreous cavity model. Collectively, these findings support further investigation of the HA-BDDE/PEG-Tyramine system as a potential vitreous substitute. Further in vivo studies will be required to establish its long-term degradation behavior, intraocular retention, retinal and ocular safety, functional performance, and overall suitability for potential clinical translation.

## 4. Materials and Methods

### 4.1. Materials

Hyaluronic acid (HA), 1,4-butanediol diglycidyl ether (BDDE), sodium hydroxide (NaOH), carboxyl-terminated poly (ethylene glycol) (PEG-COOH), 1-ethyl-3-(3-dimethylaminopropyl)carbodiimide hydrochloride (EDC), N-hydroxysuccinimide (NHS), tyramine, 2-(N-morpholino)ethanesulfonic acid (MES), phosphate-buffered saline (PBS), and deuterium oxide (D_2_O) were obtained from Sigma-Aldrich, St. Louis, MO, USA. Cell-culture reagents, including Dulbecco’s modified Eagle medium (DMEM), methyl thiazolyl tetrazolium (MTT) reagent, and Live/Dead staining reagents, were obtained from Gibco, Thermo Fisher Scientific, Waltham, MA, USA. All reagents were used as received without further purification. Deionized water was used for hydrogel preparation and solution preparation.

### 4.2. Study Design

This was an ex vivo experimental study using freshly harvested porcine eyes to evaluate the feasibility, retention, transparency, and gross structural integrity of an injectable hydrogel vitreous substitute following pars plana vitrectomy.

### 4.3. Preparation of HA-BDDE Hydrogel (Hydrogel I)

For preparation of the HA-based hydrogel, a 2% (*w*/*v*) hyaluronic acid (HA) solution was prepared in deionized water. The solution was adjusted to an alkaline pH of 11–13 using NaOH to promote the reaction between HA functional groups and the epoxide functionalities of BDDE. Subsequently, BDDE was added at a concentration of 0.5% (*v*/*v*), and the mixture was incubated overnight at room temperature to allow chemical crosslinking of the HA network. Following crosslinking, the resulting HA-BDDE hydrogel was neutralized to physiological pH (6.8–7.4) and purified by dialysis against PBS for 72 h to remove residual crosslinking reagent and low-molecular-weight soluble species. The purified HA-BDDE hydrogel was subsequently lyophilized and stored until further use.

### 4.4. Preparation of PEG-Tyramine (Hydrogel II)

PEG-Tyramine (PEG-TA) was synthesized through carbodiimide-mediated coupling of tyramine to carboxyl-terminated poly(ethylene glycol) (PEG-COOH) using EDC/NHS chemistry. Briefly, PEG-COOH was dissolved in MES buffer under mildly acidic conditions (pH 6.0–6.5), followed by the addition of EDC and NHS to activate the terminal carboxyl groups. Tyramine was subsequently added to the activated PEG solution, and the reaction mixture was maintained under continuous stirring at room temperature for 24 h. Following completion of the coupling reaction, the reaction mixture was adjusted to near-neutral pH and purified by dialysis against phosphate-buffered saline (PBS) for 72 h to remove unreacted reagents and low-molecular-weight reaction by-products. The purified PEG-TA solution was subsequently lyophilized and stored until further use.

### 4.5. Preparation of the HA-BDDE/PEG-Tyramine Dual (Hybrid)-Hydrogel

The final dual-hydrogel formulation was prepared by combining the separately synthesized and purified HA-BDDE (Hydrogel I) and PEG-Tyramine (Hydrogel II) components at a 4:1 (*w*/*w*) ratio. Importantly, the two hydrogel components were prepared and purified in advance and stored separately prior to use; thus, no polymer synthesis or crosslinking reaction was required at the time of administration. Immediately before application, the preformed components were combined at the predetermined 4:1 (*w*/*w*) ratio to obtain the final homogeneous hydrogel formulation.

### 4.6. Hydrogel Characterization

The HA-BDDE/PEG-Tyramine dual-hydrogel was characterized using complementary spectroscopic, rheological, optical, and handling-property analyses to assess its chemical composition and physicochemical characteristics relevant to its potential application as a vitreous substitute.

### 4.7. Rheological Properties

The viscoelastic properties of the HA-BDDE/PEG-Tyramine hydrogel were evaluated using oscillatory rheometry with an MCR 302 rheometer (Anton Paar GmbH, Graz, Austria). Frequency-sweep measurements were performed over 0.1–100 Hz to determine the storage modulus (G′) and loss modulus (G″) and to evaluate the frequency dependence of the hydrogel’s viscoelastic response. The relationship between G′ and G″ was used to assess the predominant elastic or viscous character of the hydrogel under dynamic deformation. Complex viscosity (η*) was also evaluated as a function of frequency over the same range. Because these measurements were performed using an oscillatory frequency sweep, the resulting viscosity profile was interpreted as frequency-dependent complex-viscosity behavior rather than as a direct measurement of steady-shear viscosity or shear-thinning [25].

### 4.8. ATR-FTIR Characterization

ATR-FTIR spectra were recorded using a FTIR spectrometer equipped with Bruker Optics GmbH & Co. KG, Ettlingen, Germany). The hydrogel sample was analyzed over the wavenumber range of 4000–400 cm^−1^. The resulting spectrum was examined to identify characteristic vibrational bands associated with hydroxyl, amine/amide, aliphatic C-H, carboxylate, and ether-containing functionalities present within the HA- and PEG-based hydrogel system [26].

### 4.9. 1H NMR Characterization

^1^H NMR spectra were acquired using a [BRAND/MODEL] NMR spectrometer operating at 600 MHz (Bruker BioSpin Corporation, Billerica, MA, USA). The molecular characteristics of the final HA-BDDE/PEG-Tyramine dual-hydrogel were further investigated by 1H nuclear magnetic resonance (1H NMR) spectroscopy. The purified hydrogel was dissolved or dispersed in D_2_O prior to analysis, and the spectrum was acquired over the relevant proton chemical-shift range. Chemical shifts were referenced to the residual solvent signal.

The 1H NMR spectrum was analyzed to identify proton environments associated with the HA and PEG components and the tyramine-derived moiety. Signals in the region of approximately 3.4–4.0 ppm were evaluated in relation to overlapping oxygenated proton environments arising from the PEG backbone and HA repeating units, while the resonance near 2.0 ppm was assigned to the methyl protons of the N-acetyl groups of HA [27,28]. The strong resonance near 4.7 ppm was attributed predominantly to residual H_2_O/HOD in the D_2_O medium. The analysis was used primarily to assess molecular composition and characteristic proton environments rather than to quantitatively determine crosslinking or residual epoxide conversion.

### 4.10. Optical Transparency

Optical transmittance was measured using a Molecular Devices SpectraMax iD microplate reader across the visible-light wavelength range of 400–700 nm. Measurements were performed at selected wavelengths of 400, 500, 600, and 700 nm using a 96-well microplate. For each measurement, 100 µL of sample was loaded into an individual well. Five independent hydrogel samples and five native porcine vitreous samples (n = 5 per group) were analyzed, and each sample was measured in triplicate under identical experimental conditions. A blank was used as the 100% transmittance reference.

Fresh porcine eyes were obtained from a local abattoir and transported to the laboratory on ice, as described in the ex vivo experimental section. All experiments were initiated within 5 h of enucleation. For optical comparison, native vitreous was carefully isolated from the porcine globes and collected without dilution. An aliquot of native vitreous was transferred directly into the microplate wells at the same volume used for the hydrogel samples and analyzed under the same experimental conditions. The optical properties of the HA-BDDE/PEG-Tyramine hydrogel were evaluated by spectrophotometric measurement across the visible-light wavelength range of 400–700 nm. The resulting transmittance values were used to generate wavelength-dependent transmission profiles and to compare the optical transmission of the hydrogel with that of native porcine vitreous [10].

### 4.11. MTT Cell Viability Assay

The cytocompatibility of the hydrogel was evaluated using a standard MTT assay. Retinal pigment epithelial (RPE) cells were obtained from the American Type Culture Collection (ATCC, Manassas, VA, USA) and cultured in complete medium as previously described [29]. The cells were cultured in DMEM supplemented with 10% fetal bovine serum (FBS) under standard culture conditions (37 °C, 5% CO_2_, and a humidified atmosphere).

For hydrogel preparation, 0.25 mL of hydrogel was placed in a sterile square silicone mold (1 × 1 cm) and allowed to form for 24 h. The resulting hydrogel disks were then transferred to 24-well culture plates. A total of 40,000 RPE cells were seeded onto each hydrogel disk. Cells cultured directly on tissue-culture-treated plates without hydrogel served as the control group. Cells were maintained under standard culture conditions (37 °C, 5% CO_2_, and a humidified atmosphere) throughout the experimental period. Cell viability was assessed after 1, 3, and 7 days of culture according to the manufacturer’s MTT protocol. Absorbance was measured at 570 nm, with 630 nm used as the reference wavelength for background correction. Cell viability was expressed as a percentage relative to the control group.

### 4.12. Live/Dead Cell Viability Assay

Cell viability and cytotoxicity were further evaluated using a Live/Dead fluorescence assay at 1, 3, and 7 days. RPE cells were seeded onto sterile hydrogel disks prepared as described above at a density of 40,000 cells per disk. Cells were maintained in DMEM supplemented with 10% FBS under standard culture conditions (37 °C, 5% CO_2_, and a humidified atmosphere).

At each time point, the hydrogel samples were gently washed with PBS and stained with a Live/Dead staining solution containing calcein-AM (3 µL/mL) to identify viable cells and propidium iodide (PI, 4 µL/mL) to identify nonviable cells, following the manufacturer’s instructions. After staining, the samples were examined using a fluorescence microscope. Viable cells exhibited green fluorescence, whereas nonviable cells exhibited red fluorescence. Cellular viability was qualitatively assessed by comparing the fluorescence staining patterns between the hydrogel and hydrogel-free control groups [30].

### 4.13. Ex Vivo Porcine Vitrectomy Model

Fresh porcine eyes were obtained from a local abattoir and transported to the laboratory on ice. All experiments were initiated within 5 h of enucleation to minimize postmortem tissue degradation. Extraocular tissues were carefully removed, and the globes were inspected for gross abnormalities. Eyes with corneal opacity, scleral rupture, retinal damage, or other apparent structural abnormalities were excluded. The intact globes were secured in a custom-made holder under an ophthalmic operating microscope. A three-port, 23-gauge pars plana vitrectomy was performed on the intact porcine globes (n = 10). Following core vitrectomy, 0.1 mL of the hydrogel vitreous substitute was injected into the vitreous cavity through a 23-gauge needle. The sclerotomy sites were subsequently sealed, and the treated globes were placed in DMEM and maintained at room temperature for 1 week. At the end of the 1-week observation period, the globes were opened for gross examination. The hydrogel was qualitatively assessed for gross appearance, optical transparency, structural integrity, and retention within the vitreous cavity.

### 4.14. Statistical Analysis

Data were analyzed using GraphPad Prism 10 (GraphPad Software, Inc., San Diego, CA, USA). Continuous variables were expressed as mean ± standard deviation. For datasets involving comparisons among multiple groups, statistical differences were evaluated using one-way analysis of variance (ANOVA) followed by Tukey’s post hoc test. A *p*-value < 0.05 was considered statistically significant.

## Data Availability

The original contributions presented in this study are included in the article. Further inquiries can be directed to the corresponding authors.
